# Reducing Phenanthrene Contamination in *Trifolium repens* L. With Root-Associated Phenanthrene-Degrading Bacterium *Diaphorobacter* sp. Phe15

**DOI:** 10.3389/fmicb.2021.792698

**Published:** 2021-11-26

**Authors:** Hui Zhao, Yujun Gu, Xiangyu Liu, Juan Liu, Michael Gatheru Waigi

**Affiliations:** College of Resources and Environmental Sciences, Institute of Organic Contaminant Control and Soil Remediation, Nanjing Agricultural University, Nanjing, China

**Keywords:** polycyclic aromatic hydrocarbons (PAHs), phenanthrene biodegradation, root-associated bacteria, colonization and distribution, plant uptake and accumulation

## Abstract

Some root-associated bacteria could degrade polycyclic aromatic hydrocarbons (PAHs) in contaminated soil; however, their dynamic distribution and performance on root surface and in inner plant tissues are still unclear. In this study, greenhouse container experiments were conducted by inoculating the phenanthrene-degrading bacterium *Diaphorobacter* sp. Phe15, which was isolated from root surfaces of healthy plants contaminated with PAHs, with the white clover (*Trifolium repens* L.) *via* root irrigation or seed soaking. The dynamic colonization, distribution, and performance of Phe15 in white clover were investigated. Strain Phe15 could efficiently degrade phenanthrene in shaking flasks and produce IAA and siderophore. After cultivation for 30, 40, and 50 days, it could colonize the root surface of white clover by forming aggregates and enter its inner tissues *via* root irrigation or seed soaking. The number of strain Phe15 colonized on the white clover root surfaces was the highest, reaching 6.03 Log CFU⋅g^–1^ FW, followed by that in the roots and the least in the shoots. Colonization of Phe15 significantly reduced the contents of phenanthrene in white clover; the contents of phenanthrene in Phe15-inoculated plants roots and shoots were reduced by 29.92–43.16 and 41.36–51.29%, respectively, compared with the Phe15-free treatment. The Phe15 colonization also significantly enhanced the phenanthrene removal from rhizosphere soil. The colonization and performance of strain Phe15 in white clove inoculated *via* root inoculation were better than seed soaking. This study provides the technical support and the resource of strains for reducing the plant PAH pollution in PAH-contaminated areas.

## Introduction

Polycyclic aromatic hydrocarbons (PAHs) are a class of persistent toxic organic pollutants that exist widely in the soil environment (Falciglia et al., 2016). PAHs are mainly derived from the incomplete combustion of petroleum, coal, wood, and other organic matter (Abdel-Shafy and Mansour, 2016). Moreover, studies have shown that the main source of PAHs has changed from the incomplete combustion of biomass to the use of large amounts of fossil fuels (Li et al., 2021; Yu et al., 2021). It can be seen that man-made production activities have become the main source of PAHs released into the environment. In Delhi, India, the total PAH contents hidden in PM_10_ and PM_2_._5_ in winter were as high as 177.5 ng⋅m^–3^, and these PAHs would enter the soil eventually (Singh et al., 2011). A study performed in 2011 found that the petroleum refining soil in Bratislava contained up to 2000 μg⋅Kg^−1^ of PAHs (Musa Bandowe et al., 2011). And to make matters worse, recent studies have shown an increase in PAHs over the Arctic (Yu et al., 2019). PAHs in the soil can be taken up and accumulated by plants and then transferred and biomagnified through the food chains, threatening human health (Gao et al., 2013). It is of great significance to regulate the absorption and accumulation of PAHs in soil by plants, to reduce the PAH contamination in crops and produce safe agricultural products in PAH-contaminated areas.

Some surfactants can enhance the solubility and bioavailability of PAHs, and are used to regulate PAH uptake and accumulation in plants. Gao et al. (2006) founded that the addition of appropriate BRIJ35 (≤74 mg⋅L^–1^) could improve the absorption of pyrene and phenanthrene by plants, while the high concentration BRIJ35 (≥148 mg⋅L^–1^) inhibited the absorption of them due to its competitive effect and toxic effect on plants. Coincidentally, Liao et al. (2015) also pointed out that adding an appropriate concentration of surfactants to the soil could improve the bioavailability of PAHs, thus promoting the absorption and accumulation of PAHs by maize. However, due to environmentally unfriendly characteristics of most surfactants (Mueller et al., 2005), microbial methods such as endophytic bacteria and mycorrhizal fungi were more commonly used to remove PAHs from rhizosphere soil and inner plants, thus reducing the risk of plant PAH pollution in contaminated areas (Gao et al., 2011; Sun et al., 2014).

The root surface is the interface between plant roots and soil, and it is an important window for plants to absorb organic pollutants. According to Fismes et al. (2002), firstly, organic pollutants gradually spread from the surrounding environment to plant roots and were adsorbed on the root surface and then gradually absorbed by plant roots. Zhan et al. (2010) verified this conclusion when studying the absorption of phenanthrene by wheat roots: in the initial stage of rapid phenanthrene absorption by wheat roots, phenanthrene was first adsorbed to the surface of wheat roots then spread to the inner root tissue and transferred to the shoots along with transpiration flow. It is clear that reducing PAH contents adsorbed by root surfaces can effectively inhibit the uptake of PAHs by plant roots, thus reducing the plant PAH contamination.

Root exudates can attract considerable numbers of bacteria to colonize the root surface of plants and form bacterial aggregates or even bacterial biofilms (Ramey et al., 2004). Root-associated bacteria usually own various ecological functions, such as promoting plant growth, improving plant stress resistance to harsh environments, reducing plant diseases (Thimmaraju et al., 2008), and controlling even remediating environmental organic pollution (Marta et al., 2005). In 2004, Johnsen and Karlson (2004) found that three strains belonging to *Sphingomonas* could increase the solubility of PAHs *via* producing exopolysaccharides, thus enhancing the degradation efficiency of PAHs. Böltner et al. (2008) isolated two *Sphingomonas* strains with lindane-degrading ability from plant root surfaces and found that the two strains colonized maize roots in large numbers for a long time and could remove lindane consistently from the rhizosphere. However, there are few reports on root-associated PAH-degrading bacteria; whether their colonization on root surface can effectively reduce the accumulation and absorption of PAHs in plants remains unclear.

The PAH-polluted agricultural area exists in many countries; it is of great significance to reuse these contaminated fields to produce safe agricultural products. In these PAH-polluted soil, phenanthrene is always one of the dominant PAHs and can be absorbed by plants (Tao et al., 2004). Based on this, this study explores the isolation and recolonization of a root-associated phenanthrene-degrading bacterium into white clover *via* root inoculation or seed soaking. The degradation performance of phenanthrene, a model PAH, by this strain was also examined. The colonization, distribution and performance of this strain in white clover were investigated to provide a theoretical basis for the plant PAH pollution control and agricultural food safety in PAH-contaminated areas.

## Materials and Methods

### Reagents and Culture Media

The phenanthrene (purity ≥98%) was purchased from Fluka, Germany, and it was prepared into a highly concentrated stock solution (2.0 g⋅L^–1^ in acetone). The water solubility (Sw) and molecular weight (MW) of phenanthrene is 1.18 mg⋅L^–1^ (25°C) and 178.23, respectively, and the logarithmic transformation distribution coefficient (Log K_ow_) of phenanthrene octanol-water is 4.57 (Chiou et al., 1998). All solvents and reagents used were of analytical grade.

The mineral salt medium (MSM) contained 0.20 g⋅L^–1^ MgSO_4_⋅7H_2_O, 1.50 g⋅L^–1^ (NH_4_)_2_SO_4_, 1.91 g⋅L^–1^ K_2_HPO_4_⋅3H_2_O and 0.50 g⋅L^–1^ KH_2_PO_4_ (Sun et al., 2014). Phenanthrene was added to MSM to make PMM medium as described in a previous study (Liu et al., 2014). Solid medium plates were prepared by adding 18 g⋅L^–1^ of agar into the liquid media described above.

### Isolation of the Root-Associated Phenanthrene-Degrading Bacterium Phe15

Several representative dominant plants were collected from the fields contaminated by PAHs for a long time. After removal of soil from the root surface with a sterilizing brush, the root was washed gently under running water for 15 s, cut off with sterilizing scissors, and then placed into a centrifuge tube with 5 mL sterilized deionized water to vibrate violently for 30 s, to destroy the bacterial biofilm structure and free the bacteria from the root surface (Yamaga et al., 2010).

The obtained bacterial suspension was added into PMM with the initial phenanthrene concentration of 100 mg⋅L^–1^ and cultivated (Liu et al., 2018). Finally, the enrichment culture was coated on PMM plates, and a bacterium named Phe15 was isolated for it could produce a clear zone around its colony. After verifying the phenanthrene-degrading ability of this strain, it was identified as mentioned previously (Sun et al., 2014).

### Degradation of Phenanthrene by Strain Phe15

Strain Phe15 was inoculated in LB medium and cultured on a rotary shaker (180 r⋅min^–1^, 30°C) until OD_600nm_ = 1.0. The bacterial solution was centrifuged (8000 r⋅min^–1^, 5 min), washed twice by MSM, resuspended to obtain 1.8 × 10^8^ colony-forming units (cfu) ⋅ mL^–1^, and injected into PMM at 2% (V: V).

Then, each 20 mL of the above inoculated PMM (containing 100 mg⋅L^–1^ phenanthrene) was added into a 50 mL flask and cultured in a shaker (180 r⋅min^–1^, 30°C). The cultured samples were extracted periodically. The Phe15 cells in the flask were estimated by plate counting, and the residual phenanthrene concentration was determined by HPLC (Liu et al., 2018). Phenanthrene degradation kinetics and growth curves of Phe15 were plotted with the PMM inoculated with inactivated Phe15 as control.

The effects of pH (4.0, 5.0, 6.0, 7.0, 8.0, 9.0, 10.0), phenanthrene concentration (50, 100, 150, 200, 250 mg⋅L^–1^) and the incubation temperature (15, 20, 25, 30, 37, 42°C) on degradation of phenanthrene by strain Phe15 were also investigated (Sun et al., 2014).

### Biological Characteristics of Strain Phe15

#### Antibiotic Resistance

The distribution of strain Phe15 in the inner and rhizosphere of plants could be tracked after antibiotic resistance label as following. The inoculum of strain Phe15 as above was inoculated in LB liquid medium containing different concentrations of antibiotics at 2% (V:V), and the growth of the strains was observed after 96 h of constant temperature oscillation culture (180 r⋅min^–1^, 30°C). Antibiotics include ampicillin, gentamicin, kanamycin, streptomycin, tetracycline, chloramphenicol, erythromycin, and spectinomycin at concentrations of 0, 10, 25, 50, 75, and 100 mg⋅L^–1^, respectively.

#### Plant Growth-Promoting Characteristics

In order to determine the plant growth promoting potential of strain Phe15, the capacity of strain Phe15 to produce indole-acetic acid (IAA) and siderophore was measured according to the methods described by Gordon and Weber (1951) and Zhang et al. (2021), respectively. Meanwhile, Phe15 pure culture (1 μL) was inoculated on the plate with LB medium supplemented with Ca_5_ (PO_4_)_3_OH and cultured at 30°C for 7 consecutive days. The appearance of transparent “halos” was observed to quantify the solubilizing effect of phosphate (Katznelson and Bose, 1959).

#### Colonization Potential on White Clover Root Surfaces

The seeds of *Trifolium repens* L. were purchased from Jiangsu Agricultural Science and Seed Industry Research Institute Co. Ltd. After using 1% sodium hypochlorite to disinfect the surface of plants for 10 min, the white clover seeds were immediately cleaned with sterile water to accelerate germination without light.

To determine the colonization ability of strain Phe15 on white clover root surfaces, the distribution of strain Phe15 on the white clover root surfaces was observed. After germination, the white clover was transferred to a sterile culture system (with 1/2 Hoagland nutrient solution) and cultured in a light incubator (Sun et al., 2014). When white clover grew to about 7 cm height, 2% (V: V) of Phe15 suspension (OD_600nm_ = 1.0) was inoculated into nutrient solution for 3 days. Then the white clover was collected, and the free cells of strain Phe15 on the white clover root surfaces were gently washed off with running water. The bacterial aggregates of Phe15 on the white clover root surfaces were observed through scanning electron microscopy (SEM) detection in the Life Science Experiment Center, Nanjing Agricultural University.

### Greenhouse Container Experiments

#### Collection and Treatment of Soil

The soil samples were collected from the topsoil of a farmland in Nanjing. The soil type was yellow-brown soil with pH value of 6.03 and organic carbon content of 14.3 g⋅kg^–1^, no PAHs were detected. The collected soil was air-dried, sieved through 10 meshes, and then artificially polluted with phenanthrene at a final content of 100 mg⋅kg^–1^ (Liu et al., 2017). After 30 days of aging (Hwang and Cutright, 2002), the phenanthrene content in the soil decreased to 62.37 mg⋅kg^–1^.

#### Potted Experiment Setup

Six treatments were set in the experiment, which were: uncontaminated soil + white clover (UW), contaminated soil (CK), contaminated soil + white clover (CW), contaminated soil + Phe15 (CP), contaminated soil + white clover + Phe15 *via* root irrigation (CWR) and contaminated soil + white clover + Phe15 *via* seed soaking (CWS). All treatments were replicated 3 times.

#### Greenhouse Container Experiments

After sterilizing the white clover seeds with 1% sodium hypochlorite for 10 min and then washing them with sterile water for 5 min, the white clover seeds were divided into two parts and one was planted directly in the soil (UW, CW, CWR). The other seeds were soaked in a prepared Phe15 MSM suspension (OD_600nm_ = 1.0) for 4 h, dried, and then sown in the soil (CWS). Follow-up treatments for the CWR group: 10 mL Phe15 suspension (OD_600nm_ = 1.0) was evenly injected into the contaminated soil when two true leaves of white clover grew after sowing. The same Phe15 suspension was also added to the unsown contaminated soil as group CP. Then, the inactivated Phe15 suspension in MSM was inoculated into CK, CW, and UW groups as the control.

Each of the six experimental groups had three pots, and each pot was evenly distributed with 8 grains. The seedlings were placed in a light incubator (25°C in the day and 20°C in the night) and cultivated for 50 days; the positions of the pots were randomly exchanged every 3 days. Destructive sampling was performed at the 30, 40 and 50 days, respectively, to determine the cell counts of colonized Phe15, the biomass of white clover, and the phenanthrene contents in plant and soil samples.

#### Determination of Biomass of White Clover

The samples of fresh white clover were collected and washed with sterile running water. After rinsing all surface attachments and drying the plant surface with blotting paper, the root and stem of white clover were separated, and the fresh weight of white clover was weighed with a balance. After that, the fresh plant samples were placed in a freeze-dryer and then freeze-dried for 72 h to obtain the dry weight.

#### Determination of Cell Count of Strain Phe15

A small number of samples (soil, plant shoots, plant roots) were ground, then suspended in sterile water and shaken to obtain bacterial suspension, which was used for dilution coating plate counting (Liu et al., 2018) on PMM plates containing 25 mg⋅L^–1^ chloramphenicol and 75 mg⋅L^–1^ ampicillin. Then, strains producing clear zones under the UV lamp were counted and then randomly selected for 16S rRNA gene sequencing. The sequencing results suggested that all the selected colonies were determined to be strain Phe15.

#### Determination of Phenanthrene Content in Soil and White Clover

A certain amount of freeze-dried samples (soil and white clover) were ground, homogenized extracted and analyzed for the phenanthrene residues by HPLC (Gao et al., 2017). The recovery of phenanthrene in the soil and plant samples averaged ≥93.8% (*n* = 5, RSD ≤ 2.47%) after the entire procedure.

### Statistical Analysis

SPSS 22.0 and Microsoft Excel 2016 software packages were used for data processing and analysis. The data point is the average of at least three repetitions; the difference was considered significant when *p*-value < 0.05, and the error bar represented standard deviation (SD).

## Results and Discussion

### Isolation and Identification of Strain Phe15

**FIGURE 1:**
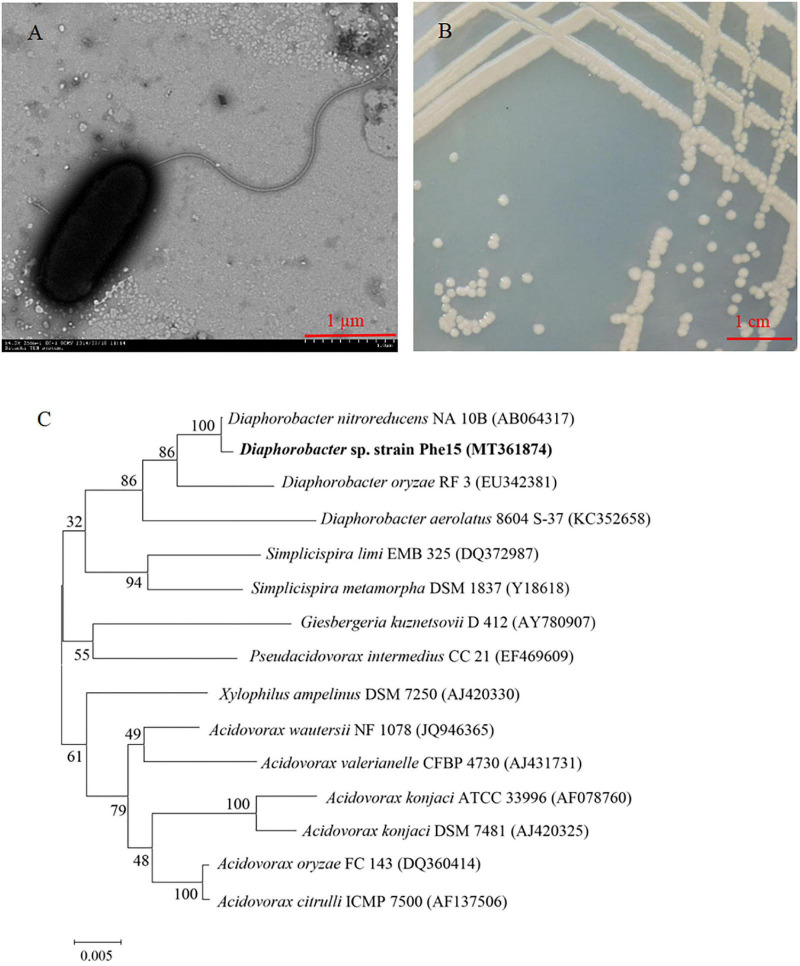
The identification of strain Phe15. **(A)** Transmission electron micrograph of strain Phe15 (× 4.0 k Zoom^–1^ HC^–1^ 80 kV); **(B)** Colonial morphology of strain Phe15 on LB agar plate; **(C)** Phylogenetic analysis of strain Phe15 and related species using the neighbor joining method. Bootstrap values (%) are indicated at the nodes in a bootstrap analysis of 1000 replicates. The scale bar indicates 0.005 changes per nucleotide. The Gen Bank accession number for each bacterium used in the analysis is shown in parentheses after the species name.

A Gram-negative rod-shaped strain Phe15, with phenanthrene-degrading function, was isolated from *Eleusine indica* (L.) Gaertn root surfaces. This strain has a terminal flagellum and its volume is about 1.8 × 0.7 μm (Figure 1A). The colonies of strain Phe15 are milky white with neat edges and smooth and moist surfaces (Figure 1B). The biochemical and physiological characteristics of Phe15 were determined and listed in Table 1. The 16S rRNA gene sequence of Phe15 (with GenBank No. of MT361874) shows more than 99% homology with that of strains belonging to *Diaphorobacter* sp. (Figure 1C). The combined results of morphology, 16S rRNA gene sequence and biochemical and physiological characteristics indicated that strain Phe15 belonged to *Diaphorobacter* sp.

**TABLE 1:** Physiological and biochemical characteristics of strain Phe15.

Test	Result	Test	Result	Test	Result
urease	+	citric acid fermentation	−	malic acid fermentation	+
indole	+	glucose fermentation	−	gram staining	−
lactose fermentation	−	glucose acidification	−	nitrate reductase	+
decanoic acid fermentation	−	phenylacetic acid fermentation	−	N-acetyl-glucosamine fermentation	−
mannose ferments	−	arabinose fermentation	+	arginine dihydrolase	+
mannitol ferments	−	β-glucosidase	−	Adipic acid fermentation	+
maltose ferments	−	gelatin liquefaction	−		

*“+” means positive; “−” means negative.*

*Diaphorobacter* sp. strains are widely found in water, soil, and sediment, it also accounted for 10% of the bacterial richness in indoor air (Miletto and Lindow, 2015). Diverse strains belonging to *Diaphorobacter* sp. could carry out simultaneous nitrification and denitrification under aerobic conditions, demonstrating a good potential for application in treatment of wastewater containing high nitrogen (Khardenavis et al., 2007). Furthermore, *Diaphorobacter* sp. strains also showed the ability to degrade various organic pollutants, such as 3-nitrotoluene (Singh and Ramanathan, 2013), 3, 4-dichloronitrobenzene (Gao et al., 2021), chloroaniline (Zhang et al., 2009), polyester polymer (Khan and Hiraishi, 2002; Qiu et al., 2015) and chlorphenuron herbicide (Zhang et al., 2018), indicating that these strains had a broad spectrum of organic pollutant degradation.

In addition, some *Diaphorobacter* sp. strains could also degrade PAHs. As early as 2009, Klankeo et al. (2009) isolated a strain, *Diaphorobacter* sp. KOTLB, from contaminated soil, which could degrade pyrene, phenanthrene, and anthracene effectively under experimental conditions. In 2016, Zhao et al. (2016) found the *Diaphorobacter* sp. contributed most of the dehydrogenases involved in PAH degradation in a cooperative metabolic network for fluoranthene degradation in polluted soil. Recently, strain *Diaphorobacter* sp. YM-6 was isolated from PAH-contaminated sediment and could degrade phenanthrene efficiently; it could degrade 96.3% of phenanthrene (with initial concentration of 100 mg⋅L^–1^) in liquid cultures within 52 h *via* phthalic acid pathway (Wang et al., 2020). In this study, strain Phe15 was isolated from the root surface of the PAH-contaminated plant, which is the first reported root-associated *Diaphorobacter* sp. strain with PAH-degrading ability.

### Degradation of Phenanthrene by *Diaphorobacter* sp. Phe15

**FIGURE 2:**
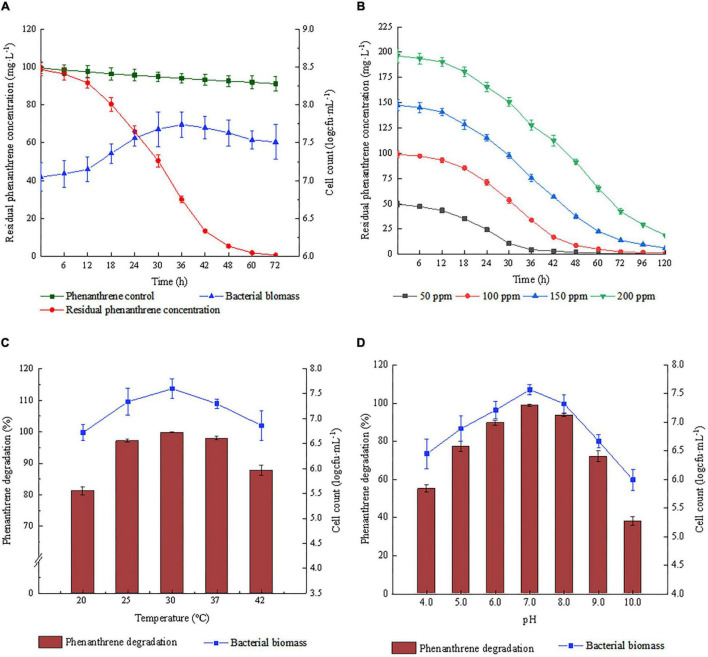
Degradation of phenanthrene by strain Phe15. **(A)** Degradation dynamics of phenanthrene and the growth curve of strain Phe15; **(B)** Effect of substrate concentration on phenanthrene degradation by strain Phe15; **(C)** Effect of temperature on phenanthrene degradation and cell growth of strain Phe15; **(D)** Effect of initial pH value on phenanthrene degradation and cell growth of strain Phe15. Error bars are standard deviations (SD), *n* = 3.

The dynamics of phenanthrene degradation by strain Phe15 were studied in liquid PMM. As shown in Figure 2A, more than 70% of the phenanthrene was degraded within 36 h and over 95% was degraded within 48 h in PMM. At the same time, the cell count of strain Phe15 increased rapidly from 12 to 36 h of cultivation, with a maximum cell count of 7.74 (Log cfu⋅mL^–1^).

Strain Phe15 could degrade phenanthrene well at a wide range of temperatures, initial pH and phenanthrene concentrations. As shown in Figure 2B, the phenanthrene was almost completely degraded within 120 h at the concentrations of 50–200 mg⋅L^–1^. Strain Phe15 grew better in the temperature range of 20–42°C and could degrade more than 80% of phenanthrene. The optimum temperature is 30°C (Figure 2C). Meanwhile, Phe15 was highly stable under pH 5.0 to 9.0, and could degrade more than 70% of phenanthrene. The optimal pH was 7.0, and the degradation ratio was rapidly reduced to 40–50% when the pH value was lower than 4.0 or higher than 10.0 (Figure 2D).

Compared with the other PAH-degrading *Diaphorobacter* sp. strains, Phe15 demonstrated better degrading ability and higher tolerance to environmental conditions. For instance, it took 8 days for *Diaphorobacter* sp. KOTLB to fully degrade phenanthrene (with concentration of 100 mg⋅L^–1^) in liquid cultures (Klankeo et al., 2009), and 52 h for *Diaphorobacter* sp. YM-6 to degrade 96% of it (Klankeo et al., 2009); while for strain Phe15, it took 48 h to degrade 95% of it. When the pH value was below 6.0 or above 10.0, the phenanthrene degradation ratio by strain YM-6 quickly deceased to lower than 20%, while for Phe15, when the pH value was between 4.0–10.0, the degradation ratio was all higher than 40%. Obviously, strain Phe15 showed better potential for application in practical use in environmental remediation.

### Biological Characteristics of *Diaphorobacter* sp. Phe15

Strain Phe15 was resistant to 25 mg⋅L^–1^ chloramphenicol and 75 mg⋅L^–1^ ampicillin, its distribution in the rhizosphere and inner plant tissues could be tracked by using the resistance to these two antibiotics as markers. Antibiotic markers, as well as the *lacZ, xylE*, and *lux* gene markers, are commonly used to track the bacterial environmental behaviors, and beneficial to the screening and counting of target strains (Prosser, 1994). For example, the ecological behavior of *Pseudomonas* mutants in organic soils was successfully monitored based on their resistance to rifampicin (Compeau et al., 1988). Furthermore, the environmental risk of fluoroquinolones was carefully evaluated *via* monitoring the resistance of *Streptococcus pneumoniae* to ciprofloxacin and other drugs (Sahm et al., 2000). These studies suggest that antibiotic markers are also a good option if wild strains are not suitable for genetic modification to add genetic markers.

Strain Phe15 could produce 15.89 mg⋅L^–1^ IAA in nitrogen medium and had a specific capacity for producing siderophores. It has been found that some nitrogen-fixing bacteria isolated from tropical grass species can easily colonize plants by producing certain factors that promote plant growth (Lugtenberg and Kamilova, 2009). Similarly, the bacteria inhibiting the growth of fungal pathogens by producing siderophores, have a high advantage in rhizosphere nutrition and niche competition (Schippers et al., 1987). These rhizosphere bacteria, which have mechanisms such as nitrogen fixation and phosphate dissolution, rhizosphere engineering and plant hormone production, are collectively referred as plant-promoting rhizosphere bacteria (PGPR), such as *Rhizobia* (Wang and Martínez-Romero, 2000), *Pseudomonas fluorescens* (Khalid et al., 2004) and *Bacillus subtilis* (Bhattacharyya and Jha, 2012). They can better colonize on the root surface and then migrate into inner plants.

**FIGURE 3:**
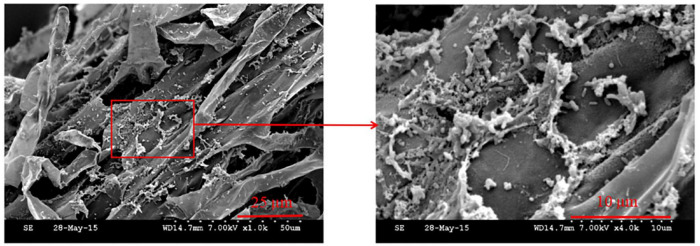
The colonization of strain Phe15 on the root surface of white clover observed by scanning electron microscope (WD14.7 mm 7.00 kV). The magnification of the left and right image is 1.0 and 4.0 k, respectively.

The results of the colonization experiment showed that Phe15 could colonize well on white clover root surfaces and form aggregates (Figure 3). Studies have shown that these aggregates are conducive to the long-term existence of rhizosphere bacteria on the root surface (Singh et al., 2006), because bacteria in the aggregates have the ability to resist adverse living environments and microbial inhibitors (Davey and O’toole, 2000) and can induce the activities of some enzymes in inner plants to remove organic pollutants such as PAHs (Shehzadi et al., 2014). Therefore, rhizosphere degrading bacteria are often combined with plants to remove organic pollutants from soil, among which root-associated bacteria that could form aggregates or biofilms on the root surface are always preferred (Khan et al., 2013; Arslan et al., 2017).

### Colonization and Distribution of *Diaphorobacter* sp. Phe15 in White Clover

In the greenhouse container experiments, after inoculation of strain Phe15 for 30–50 days, most Phe15 cells efficiently colonized on the white clover root surfaces, and a few cells entered the inner tissues of white clover or were released into the rhizosphere soil (Table 2). For instance, the number of Phe15 cells was the highest on the root surface of white clover (5.58 Log CFU⋅g^–1^ FW *via* seed soaking and 6.03 Log CFU⋅g^–1^ FW *via* root irrigation) after inoculation for 30 d in phenanthrene contaminated soil.

**TABLE 2:** The cell counts of strain Phe15 colonized on the root surface, in the inner tissues and soil after inoculation with white clover for 30, 40, and 50 d.

Treatments		Cell counts of strain Phe15 (Log CFU⋅g^–1^ FW)			
		Shoot	Root	Root surface	Soil
30d	CP	−	−	−	5.47 ± 0.21b
	CWR	4.65 ± 0.14a	5.38 ± 0.17a	6.03 ± 0.13a	5.79 ± 0.01a
	CWS	4.32 ± 0.10*bc*	5.05 ± 0.19*bc*	5.58 ± 0.05*bc*	4.85 ± 0.05c
40d	CP	−	−	−	4.51 ± 0.04*de*
	CWR	4.47 ± 0.07*ab*	5.12 ± 0.11*ab*	5.85 ± 0.17*ab*	4.89 ± 0.10c
	CWS	3.81 ± 0.11d	4.57 ± 0.06d	5.32 ± 0.34*cd*	4.70 ± 0.05*cd*
50d	CP	−	−	−	4.31 ± 0.01e
	CWR	4.22 ± 0.09c	4.87 ± 0.13c	5.55 ± 0.16*cd*	4.69 ± 0.08*cd*
	CWS	3.45 ± 0.14e	4.02 ± 0.07e	4.94 ± 0.09d	4.51 ± 0.04*de*

*CP, contaminated soil inoculated with strain Phe15; CWR, contaminated soil planted with white clover and inoculated with strain Phe15 via root irrigation; CWS, contaminated soil planted with white clover and inoculated with strain Phe15 via soaked seed; FW, fresh weight; ″−″indicates not detected. Different letters in the same column indicate significant differences (P < 0.05).*

Furthermore, with the time extended from 30 to 50 days after inoculation, the number of Phe15 cells colonized in white clover and rhizosphere soil decreased obviously (*P* < 0.05). In most cases, the cell counts of Phe15 colonized in white clover plants *via* root irrigation were significantly higher than those *via* seed soaking (*P* < 0.05, Table 2). The reason may be that the cell count of Phe15 inoculated *via* seed soaking was much less, and the inoculation time was earlier than that *via* root irrigation. In addition, the decay rate of Phe15 counts in soil was also slowed down by planting white clover, indicating that white clover could provide a favorable environment for the colonization of Phe15 and facilitate its long-term survival in the system.

There are a considerable number of bacteria in the rhizosphere environment, and some of them could colonize the root surface of plants through the selection of root exudates (Bais et al., 2006; Wang et al., 2021); among which some bacteria could enter the inner tissues of roots and become endophytic bacteria (Mesa-Marin et al., 2019). In general, plant growth-promoting bacteria isolated from the root surface are more easily recolonized on the root surface of the plant and could survive in this system for a long time (Zhu et al., 2021), which is consistent with the results of this study. Strain Phe15 was isolated from the root surface of *Eleusine indica* (L.) Gaertn and could colonize and survive well on the root surface of white clover, some Phe15 cells could also enter the root tissue and transfer to shoot, suggesting that strain Phe15 might be a good candidate for phytoremediation of PAH-contaminated soil.

### Colonization of Strain Phe15 Promoted the Growth of White Clover

As shown in Tables 3, 4, the high content of phenanthrene significantly inhibited growth of white clover during the first 40-day cultivation (*P* < 0.05), while the inoculation of Phe15 alleviated the inhibition obviously (*P* < 0.05). After cultivation for 50 days, the phenanthrene content in the soil is relatively low, basically relieving the phenanthrene stress to white clover, the inoculation of Phe15 significantly promoted the root growth of white clover *via* root irrigation (*P* < 0.05). Furthermore, compared with *via* seed soaking, more Phe15 cells colonized in white clover *via* root irrigation, demonstrating obvious advantage in relieving phenanthrene stress and promoting white clover growth.

**TABLE 3:** The root biomass of white clover in different treatments (mg⋅pot^–1^) after inoculation with strain Phe15 for 30, 40, and 50 d.

Treatments	30 d		40 d		50 d	
	Fresh weight (mg⋅pot^–1^)	Dry weight (mg⋅pot^–1^)	Fresh weight (mg⋅pot^–1^)	Dry weight (mg⋅pot^–1^)	Fresh weight (mg⋅pot^–1^)	Dry weight (mg⋅pot^–1^)
UW	25.43 ± 1.03a	5.34 ± 0.28a	125.23 ± 6.71a	23.45 ± 1.25a	185.85 ± 15.70b	35.74 ± 0.80b
CW	20.82 ± 0.76c	4.46 ± 0.10b	99.09 ± 4.08c	19.11 ± 1.49c	181.42 ± 9.67b	34.51 ± 1.79b
CWR	23.89 ± 0.78b	5.14 ± 0.21a	119.96 ± 2.54a	22.14 ± 1.32*ab*	206.43 ± 9.17a	38.28 ± 0.98a
CWS	22.70 ± 0.57b	5.26 ± 0.15a	111.17 ± 2.78b	20.50 ± 1.81*bc*	176.51 ± 3.87b	35.28 ± 0.87b

*UW, uncontaminated soil planted with white clover; CW, contaminated soil planted with white clover; CWR, contaminated soil planted with white clover and inoculated with strain Phe15 via root irrigation; CWS, contaminated soil planted with white clover and inoculated with strain Phe15 via soaked seed. Different letters in the same column indicate significant differences (P < 0.05).*

**TABLE 4:** The shoot biomass of white clover in different treatments (mg⋅pot^–1^) after inoculation with strain Phe15 for 30, 40, and 50 d.

Treatments	30 d		40 d		50 d	
	Fresh weight (mg⋅pot^–1^)	Dry weight (mg⋅pot^–1^)	Fresh weight (mg⋅pot^–1^)	Dry weight (mg⋅pot^–1^)	Fresh weight (mg⋅pot^–1^)	Dry weight (mg⋅pot^–1^)
UW	479.12 ± 8.69a	42.73 ± 1.67a	1026.86 ± 30.30a	116.30 ± 3.86a	2159.77 ± 58.23a	252.59 ± 20.93a
CW	388.36 ± 11.3c	36.12 ± 1.32c	809.83 ± 16.21d	103.07 ± 3.71b	1802.81 ± 23.97c	233.43 ± 13.23a
CWR	405.54 ± 6.48b	37.63 ± 1.66*bc*	981.78 ± 23.93b	116.01 ± 2.59a	2178.59 ± 31.76a	239.23 ± 18.92a
CWS	418.55 ± 6.55b	39.51 ± 0.90b	905.76 ± 13.70c	106.33 ± 3.52b	2031.15 ± 24.56b	237.66 ± 17.30a

*UW, uncontaminated soil planted with white clover; CW, contaminated soil planted with white clover; CWR, contaminated soil planted with white clover and inoculated with strain Phe15 via root irrigation; CWS, contaminated soil planted with white clover and inoculated with strain Phe15 via soaked seed. Different letters in the same column indicate significant differences (P < 0.05).*

Because Phe15 could degrade phenanthrene efficiently, after stable colonization on the root surface of white clover, it removed the phenanthrene from the white clover-soil system effectively and relieved the toxic stress to white clover caused by contamination. Meanwhile, Phe15 could also produce a certain amount of IAA and siderophores, significantly promoting the white clover growth after relieving the phenanthrene stress in white clover. Correspondingly, the white clover provided a stable habitat for Phe15, which could better play the role of phenanthrene degradation and plant growth promotion. Similar results were also found in previous studies (Zhou and Gao, 2019), indicating that well-constructed rhizosphere interaction between plants and bacteria could effectively remove soil organic pollutants and maintain a healthy rhizosphere environment.

### Colonization of Strain Phe15 Reduced the Phenanthrene Contents in White Clover

The colonization of strain Phe15 on the white clover root surfaces effectively reduced the contents and accumulation of phenanthrene in white clover shoots and roots (Table 5). For instance, after 40 days of cultivation, compared with the Phe15-free treatments, in Phe15-inoculated treatments, the phenanthrene contents in shoots and roots of white clover were reduced by 51.29 and 43.16% *via* root irrigation and 48.39 and 35.40% *via* seed soaking, respectively (*P* < 0.05). Correspondingly, the accumulation of phenanthrene in white clover shoots and roots also decreased. Furthermore, the transfer and enrichment factor of phenanthrene in white clover were also obviously reduced after Phe15 inoculation. Meanwhile, compared with *via* seed soaking, inoculation of Phe15 with white clover *via* root irrigation was more effective for phenanthrene removal from white clover, and the difference was more obvious with the extension of cultivation time.

**TABLE 5:** The phenanthrene content and accumulation in white clover in different treatments after inoculation with strain Phe15 for 30, 40, and 50 d.

Treatments		Phenanthrene content (mg⋅kg^–1^)		Accumulation (μ g⋅pot^–1^)		Enrichment factor (EF)		Translocation factor (TF)
		Root	Shoot	Root	Shoot	Root	Shoot	
30 d	CW	20.52 ± 1.19a	6.78 ± 0.21a	0.09	0.24	0.81	0.27	0.33
	CWR	14.38 ± 0.72b	3.68 ± 0.17b	0.07	0.13	0.63	0.16	0.26
	CWS	14.22 ± 1.02b	3.63 ± 0.21b	0.07	0.14	0.61	0.16	0.25
40 d	CW	10.82 ± 0.95c	3.10 ± 0.09c	0.21	0.31	0.93	0.26	0.29
	CWR	6.15 ± 0.11d	1.51 ± 0.05e	0.14	0.18	0.64	0.16	0.25
	CWS	6.99 ± 0.13d	1.60 ± 0.12e	0.14	0.17	0.65	0.15	0.23
50 d	CW	6.46 ± 0.44d	1.91 ± 0.05d	0.22	0.45	0.97	0.29	0.30
	CWR	3.69 ± 0.31e	0.98 ± 0.03f	0.14	0.23	0.66	0.17	0.27
	CWS	4.18 ± 0.10e	1.12 ± 0.02f	0.15	0.27	0.66	0.18	0.27

*CW, contaminated soil planted with white clover; CWR, contaminated soil planted with white clover and inoculated with strain Phe15 via root irrigation; CWS, contaminated soil planted with white clover and inoculated with strain Phe15 via soaked seed. Phenanthrene accumulation (A) was calculated as follows: A = CP × M, CP = phenanthrene content (mg⋅kg^–1^), M = dry weight (mg⋅pot^–1^). Different letters in the same column indicate significant differences (P < 0.05). Translocation factor was defined as the ratio of phenanthrene content in shoots to phenanthrene content in roots. Enrichment factor is defined as the ratio of phenanthrene content in plant to phenanthrene content in soil.*

The root surface is a window for plants to take up organic pollutants. Degrading bacteria colonizing on plant root surfaces could quickly metabolize the organic pollutants absorbed on the root surface, thus preventing them from being taken up by plant, which is an effective method to reduce the content of organic pollutants in plant (Singh et al., 2006). In addition, because some degrading bacteria colonizing on the root surface can enter into plant and become endophytic bacteria, they could degrade organic pollutants in inner plant and reduce the pollutant accumulation (Sun et al., 2014). In this study, with phenanthrene as the representative PAHs, its uptake by white clover from the soil with or without Phe15 inoculation was investigated. The results revealed that Phe15 colonization on the white clover root surfaces could effectively remove phenanthrene from the inner white clover tissues.

Bacteria in root surfaces aggregates are often wrapped in extracellular polymers secreted by them, enhancing the solubility of phenanthrene, thus, making it easier to be utilized by bacteria (Johnsen and Karlson, 2004; Seo and Bishop, 2007). Under natural conditions, bacterial aggregates on the root surface are always formed by a variety of bacteria, which usually have different metabolic abilities and can complete a series of complex metabolic processes together, thus making the metabolism of phenanthrene more convenient and fast (Singh et al., 2006). In addition, because bacteria in the aggregation on the root surface are closely bound together, the horizontal migration of functional genes between bacterial cells are more convenient, enhancing the synergistic metabolism of phenanthrene on the root surface (Singh et al., 2006; Weyens et al., 2010; Arslan et al., 2017).

### Colonization of Strain Phe15 Enhanced the Phenanthrene Removal From Soil

**FIGURE 4:**
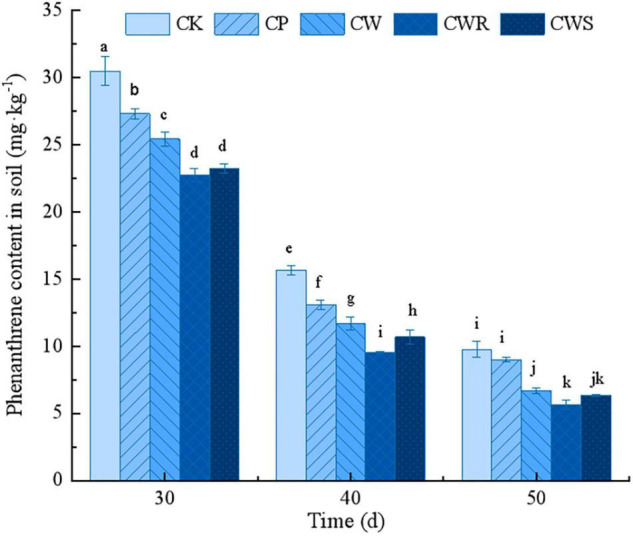
The phenanthrene residues in soil after inoculation for 30, 40, and 50 days. CK, contaminated soil; CP, contaminated soil inoculated with strain Phe15; CW, contaminated soil planted with white clover; CWR, contaminated soil planted with white clover and inoculated with strain Phe15 *via* root irrigation; CWS, contaminated soil planted with white clover and inoculated with strain Phe15 *via* soaked seed. The different lowercase letters on the bars indicate significant differences among treatments (*P* < 0.05).

Compared with the untreated phenanthrene-contaminated soil (CK), the phenanthrene contents of the soil in the treated groups were significantly reduced (*P* < 0.05). The cooperative treatment of Phe15 and white clover (CWR and CWS) was obviously better than that of only inoculated with Phe15 (CP) or only planted white clover (CW) in removing phenanthrene from soil (*P* < 0.05). For example, after cultivation for 40 days, compared with the CK group, the phenanthrene content in soil inoculating with Phe15 (CP) or planting white clover (CW) decreased by 16.37 and 25.32%, respectively; while under the combination of Phe15 and white clover *via* root irrigation or seed soaking, the phenanthrene content in soil decreased by 39.00 and 31.71%, respectively (Figure 4).

The soil environment is relatively complex, there are many inhibitors limiting the activities of the enzymes involved in PAH degradation, among which the quorum sensing mechanism has a great impact on PAH degradation efficiency (Jung et al., 2020). In the practical soil bioremediation, nutrients such as nitrogen, phosphorus, and potassium are often added to the soil environment to promote the bacterial growth and PAH degradation (Arslan et al., 2014; Premnath et al., 2021). In contrast, growing plants provide a stable habitat and root exudates as the nutrient substances for root-associated PAH-degrading bacteria, which could degrade PAHs more efficiently and persistently, and plants themselves could also metabolize PAHs, thus obtaining higher removal efficiency than that *via* single inoculation of degrading bacteria (Dai et al., 2020). The results of this study confirmed this conclusion. At the same time, with the time extension from 30 to 50 days, the residual phenanthrene content in soil decreased much as a whole, indicating that indigenous bacteria and abiotic degradation also played essential roles in the phenanthrene removal process.

## Conclusion

A root-associated phenanthrene-degrading bacterium, *Diaphorobacter* sp. Phe15, was isolated from *Eleusine indica* (L.) Gaertn and colonized on the root surface of white clover. Phe15 colonization observably reduced the phenanthrene content in white clover, promoted the white clover growth and enhanced the phenanthrene removal from soil. However, the effects of Phe15 colonization on phenanthrene metabolic enzyme activities and the molecular mechanisms involved in phenanthrene degradation in inner plants remain unclear. The richness and expression of phenanthrene-degrading genes in the soil-clover system need to be further studied. Only by supplementing and perfecting the above studies can we comprehensively and deeply analyse the mechanisms of reducing plant PAH pollution by using root-associated PAH-degrading bacteria, so as to provide a basis for the better application of this technology in practical agricultural production.

## Data Availability Statement

The datasets presented in this study can be found in online repositories. The names of the repository/repositories and accession number(s) can be found below: https://www.ncbi.nlm.nih.gov/genbank/, MT361874.

## Author Contributions

HZ conducted the experiments, analyzed the data, and wrote the manuscript. YG conducted the experiments and analyzed the data. XL analyzed the data and revised the manuscript. JL designed the study and wrote the manuscript. MW revised the manuscript. All authors contributed to the article and approved the submitted version.

## Conflict of Interest

The authors declare that the research was conducted in the absence of any commercial or financial relationships that could be construed as a potential conflict of interest.

## Publisher’s Note

All claims expressed in this article are solely those of the authors and do not necessarily represent those of their affiliated organizations, or those of the publisher, the editors and the reviewers. Any product that may be evaluated in this article, or claim that may be made by its manufacturer, is not guaranteed or endorsed by the publisher.
